# Comparative analysis of candidate gene expression in Asian atopic dermatitis and psoriasis: identification of shared and distinct molecular features

**DOI:** 10.3389/fmed.2026.1878876

**Published:** 2026-08-04

**Authors:** Hye Li Kim, Dong Eun Kim, KeLun Zhang, Su Min Kim, Sung Hee Kim, Yeon Woo Jung, Nicole Bischof, Jungmin Choi, Tae-Gyun Kim, Chang Ook Park

**Affiliations:** 1 Department of Dermatology, Severance Hospital, Cutaneous Biology Research Institute, Yonsei University College of Medicine, Seoul, Republic of Korea; 2 Brain Korea 21 PLUS Project for Medical Science, Yonsei University College of Medicine, Seoul, Republic of Korea; 3 Yonsei University College of Medicine, Seoul, Republic of Korea; 4 Department of Biomedical Sciences, Korea University College of Medicine, Seoul, Republic of Korea

**Keywords:** atopic dermatitis, filaggrin, loricrin, psoriasis, serine protease inhibitor Kazal-type 5, serpin family B member 4

## Abstract

**Background:**

Atopic dermatitis (AD) and psoriasis are chronic inflammatory skin diseases with distinct molecular profiles, but their clinical differentiation in Asian patients is often difficult due to overlapping or atypical features. This study aimed to identify differentially expressed genes (DEGs) and pathways in lesional skin to explore potential diagnostic and therapeutic targets.

**Methods:**

To identify molecular differences, we performed **t**ranscriptomic analysis of lesional skin from patients with AD, psoriasis, and healthy controls (HCs). DEGs were identified by comparing each disease group with HCs, and selected targets were validated using immunofluorescence staining.

**Results:**

Filaggrin (*FLG*) expression was significantly decreased in both the AD (*p* = 0.0043) and psoriasis (*p* = 0.0012) compared to HCs. Moreover, loricrin (*LOR*) expression was significantly reduced in both the AD (*p* = 0.0043) and psoriasis (*p* = 0.0082) relative to HCs. Serine protease inhibitor Kazal-type 5 (*SPINK5*) expression was markedly elevated in the psoriasis (*p* = 0.0012), but was significantly decreased in AD (*p* = 0.0173). Additionally, serpin family B member 4 (*SERPINB4*) expression was significantly upregulated in both AD (*p* = 0.0043) and psoriasis (*p* = 0.0012) relative to HCs. Immunofluorescence analysis of human skin samples confirmed these transcriptomic findings at the protein level.

**Conclusion:**

*FLG*, *LOR*, and *SERPINB4* showed shared expression alterations in AD and psoriasis, whereas *SPINK5* demonstrated the most consistent differential expression pattern between the two diseases. These findings suggest that *SPINK5* may represent a candidate molecular signature for distinguishing AD from psoriasis, and provide insight into shared and distinct molecular mechanisms underlying these diseases in Asian patients.

## 1 Introduction

Atopic dermatitis (AD) and psoriasis are among the most common chronic inflammatory skin diseases, both of which significantly impair the quality of life of patients. Although these diseases share some pathological mechanisms, they are characterized by distinct immunological and pathological features (1–5). Clinically, AD typically affects flexural areas and presents as thin patchy lesions with poorly defined borders. In contrast, psoriasis predominantly involves the extensor surfaces, manifesting as well-demarcated chronic plaque lesions with thick scaling. Comorbidity patterns also differ. AD is often associated with atopic comorbidities, such as asthma, food allergies, allergic rhinitis, and conjunctivitis, whereas psoriasis is more frequently associated with metabolic conditions such as hypertension and diabetes (6, 7). Immunologically, AD is mainly driven by T helper (Th)2-mediated immune responses accompanied by epidermal barrier dysfunction, such as filaggrin deficiency (8–11). In contrast, psoriasis is characterized by Th17/Th22-driven inflammation, which leads to epidermal hyperproliferation and abnormal keratinization (12–15). Despite these differences, both diseases share a common mechanism in which keratinocytes respond to T-cell-derived cytokines, altering their growth and differentiation, and thereby shaping the disease phenotype (1, 2, 16).

In European-American populations, AD and psoriasis are generally distinguished by their characteristic clinical presentations (3–5). However, studies have suggested that Asian patients with AD exhibit clinical and pathological features resembling psoriasis, raising the possibility of shared disease mechanisms (7). Therefore, a comparative transcriptomic analysis of AD and psoriasis in Asian populations is essential to elucidate disease-specific gene expression patterns and identify potential therapeutic targets.

In this study, we performed transcriptomic profiling to compare immune-related and epidermal barrier-associated gene expression differences between Asian patients with AD and psoriasis. By delineating the molecular landscape of each disease, our findings may provide insights into candidate molecular signatures and inform the development of targeted therapeutic strategies tailored to Asian populations.

## 2 Materials and methods

### 2.1 Patient recruitment and ethical approval

Patients and healthy volunteers were recruited from the Department of Dermatology at Severance Hospital, Yonsei University Health System. Patients with AD were diagnosed according to the Hanifin and Rajka criteria, and those with psoriasis were clinically diagnosed by dermatologists. All patients with psoriasis had plaque psoriasis. Skin biopsy samples from the lesional trunk were obtained from patients with AD and psoriasis. Patients with AD were evaluated using the Eczema Area and Severity Index (EASI) score, and patients with psoriasis were assessed using the Psoriasis Area and Severity Index (PASI) score. In patients with AD, total serum IgE levels and peripheral blood eosinophil counts were recorded. AD phenotype was classified as extrinsic or intrinsic according to total serum IgE levels, with levels >150 kU/L defining extrinsic AD and levels <150 kU/L defining intrinsic AD (17). Patients who had not received systemic or topical treatment for at least 4 weeks prior to biopsy were included. Patients presenting with clinical evidence of other chronic inflammatory skin diseases were excluded. All procedures involving human participants were performed according to the principles of the Declaration of Helsinki. Written informed consent was obtained from all the participants, and the study protocol was approved by the Institutional Review Board of Severance Hospital, Yonsei University College of Medicine, Seoul, South Korea (No. 4–2013-0624, 4–2017-0782).

### 2.2 Skin sample collection

A total of 39 Asian skin biopsy samples were analyzed in this study. The same samples from patients with moderate to severe AD (*n* = 6), patients with moderate to severe psoriasis (*n* = 7), and HCs (*n* = 11) were used for both transcriptomic profiling and quantitative reverse transcription PCR validation. For human skin immunofluorescence staining, biopsy specimens were obtained from a separate cohort of patients with AD (*n* = 5), psoriasis (*n* = 5), and HCs (*n* = 5). Patients’ characteristics are summarized in Supplementary Table S1.

### 2.3 Transcriptomic analysis

The AD and psoriasis transcriptomic datasets comprised independent, non-overlapping cohorts. Lesional AD skin and healthy control skin samples were analyzed by microarray, whereas lesional psoriasis skin and a separate healthy control group were analyzed by RNA sequencing.

Total RNA was isolated from human skin biopsy samples obtained from six patients with AD, seven patients with psoriasis, and 11 HCs.

Transcriptomic profiling of AD (*n* = 6), HC (*n* = 5) samples was performed using microarray analysis were conducted by Macrogen (Seoul, Korea). RNA integrity and purity were assessed using an ND-1000 spectrophotometer (NanoDrop, Wilmington, DE, USA) and an Agilent 2100 Bioanalyzer (Agilent Technologies, Palo Alto, CA, USA). A total of 400 ng RNA was reverse-transcribed using T7-oligo (dT) promoter primers. Following second-strand cDNA synthesis, the resulting cDNA was amplified and labeled using the TargetAmp Nano Labeling Kit for Illumina Expression BeadChip (Epicenter, Madison, WI, USA) to generate biotinylated cRNA according to the manufacturer’s instructions. The labeled cRNA was purified and quantified using an ND-1000 spectrophotometer. Subsequently, the labeled cRNA samples were hybridized to Human HT-12 v4.0 Expression BeadChips (Illumina Inc., San Diego, CA, USA) for 17 h at 58 °C, according to the manufacturer’s protocol. Hybridization signals were detected using Amersham fluorolink streptavidin-Cy3 (GE Healthcare Bio-Sciences, Little Chalfont, UK), and the arrays were scanned using an Illumina Bead Array Reader. Raw data were extracted using GenomeStudio software (Illumina GenomeStudio v2011.1; Gene Expression Module v1.9.0).

RNA sequencing (RNA-seq) was performed on skin biopsy samples from patients with psoriasis (*n* = 7), HCs (*n* = 6). Total RNA was extracted using the RNeasy Plus Mini Kit (Qiagen), and RNA-seq libraries were prepared with the SureSelect Strand Specific RNA Reagent Kit (Agilent Technologies), following the manufacturer’s protocol. Library quality and concentration were assessed using the Agilent 2100 BioAnalyzer (Agilent Technologies). Paired-end sequencing with a read length of 100 bp was conducted on an Illumina HiSeq2500 platform. Sequencing reads were subjected to quality control, where reads containing more than 10% ambiguous bases (N) or more than 40% low-quality bases (quality score < 20) were discarded. High-quality reads were aligned to the human reference genome using STAR (v2.4.0b). Functional annotation was performed using Gene ontology. Differential expression analysis and data visualization were conducted using R software (version 3.2.6; www.r-project.org).

Cross-platform normalization was performed to adjust for distributional differences between microarray and RNA-seq datasets. The Rank-In method, a rank-based normalization approach (18), was applied to place data from both platforms on a common scale while minimizing non-biological variation. Specifically, gene expression values within each sample were converted into rank orders to represent their relative expression levels, rather than their absolute intensities. By aligning the rank-based expression distributions across platforms, systematic differences in scale and dynamic range were reduced while preserving within sample relative expression structure. This normalized dataset was then used for the comparative analysis of four key genes across HC, AD, and psoriasis.

### 2.4 Quantitative reverse transcription PCR

Total RNA previously extracted from skin biopsy samples of patients with AD, psoriasis, and HCs for microarray and RNA-seq analysis was also used for RT-qPCR. RNA reverse transcription was carried out with the help of AccuPower RT PriMix (Bioneer), and quantitative PCR was performed with cDNA and the appropriate TaqMan primers on a StepOnePlus PCR system (Applied Biosystems). Relative gene expression levels were normalized to glyceraldehyde-3-phosphate dehydrogenase (*GAPDH*) and quantified using the 2^−ΔΔCt^ method. The primers sequences used are listed in Supplementary Table S2.

### 2.5 Immunofluorescence staining

Immunofluorescence staining was performed on 4 μm-thick cryosections of OCT-embedded AD (*n* = 5), psoriasis (*n* = 5), and HC (*n* = 5) skin samples. The tissue sections were fixed and permeabilized with 100% acetone at −20 °C for 10 min, followed by air-drying and rehydration in phosphate-buffered saline (PBS) for 10 min at room temperature. To block nonspecific binding, the sections were incubated with 5% bovine serum albumin (BSA) in PBS for 1 h at room temperature. Primary antibodies were applied overnight at 4 °C in 2% BSA. The following rabbit polyclonal primary antibodies were used: filaggrin (FLG; PA5-115235, Thermo Fisher Scientific), loricrin (LOR; PA5-30583, Thermo Fisher Scientific), serine protease inhibitor Kazal-type 5 (SPINK5; NBP1-90509; Novus Biologicals), and serine family B member 4 (SERPINB4; orb416939, Biorbyt). After washing with PBST, the sections were incubated with secondary fluorescent antibodies for 1 h at room temperature in the dark. Anti-rabbit FITC-conjugated IgG (Thermo Fisher Scientific) was used for FLG and LOR, and anti-rabbit Alexa Fluor 555-conjugated IgG (Thermo Fisher Scientific) was used for SPINK5 and SERPINB4. Finally, the tissue was mounted with VECTASHIELD™ mounting medium (Vector Laboratories, Burlingame, CA, USA) and examined using a confocal microscope LSM780 (Carl Zeiss Microscopy, Jena, Germany) at 20x magnification.

### 2.6 Statistical analysis

Statistical differences were considered significant at *p* < 0.05. Comparisons between two groups were evaluated using a two-sided Mann–Whitney U test, whereas comparisons among three groups were evaluated using the Kruskal–Wallis test followed by Dunn’s multiple comparisons test. Individual data points are shown in the graphs. Graphs were expressed as mean± standard deviation (SD) using GraphPad Prism v.8 (San Diego, CA, USA).

For transcriptomic data, differential gene expression (DEG) analysis was performed using DESeq2 package to compare gene expression profiles between AD and HC groups, and between psoriasis and HC groups. Genes with an adjusted *p*-value <0.05 and |log₂ fold change| > 2 were considered significantly differentially expressed. Principal component analysis (PCA) was used to assess overall transcriptional variance and sample clustering. To assess potential platform-associated clustering, PCA was performed before and after cross-platform normalization using platform annotations. Silhouette scores were calculated based on transcriptomic platform labels to quantitatively evaluate platform-associated separation before and after normalization. These statistical analyses were conducted using R software. DEGs were visualized using heatmaps and volcano plot generated in R and GraphPad Prism v.8.

## 3 Results

### 3.1 Key DEGs in AD skin and psoriasis skin

Transcriptomic differences between six moderate-to-severe AD samples and seven moderate-to-severe psoriasis samples were analyzed by comparing their gene expression profiles with those of 11 healthy control (HC) skin samples.

**Figure 1:**
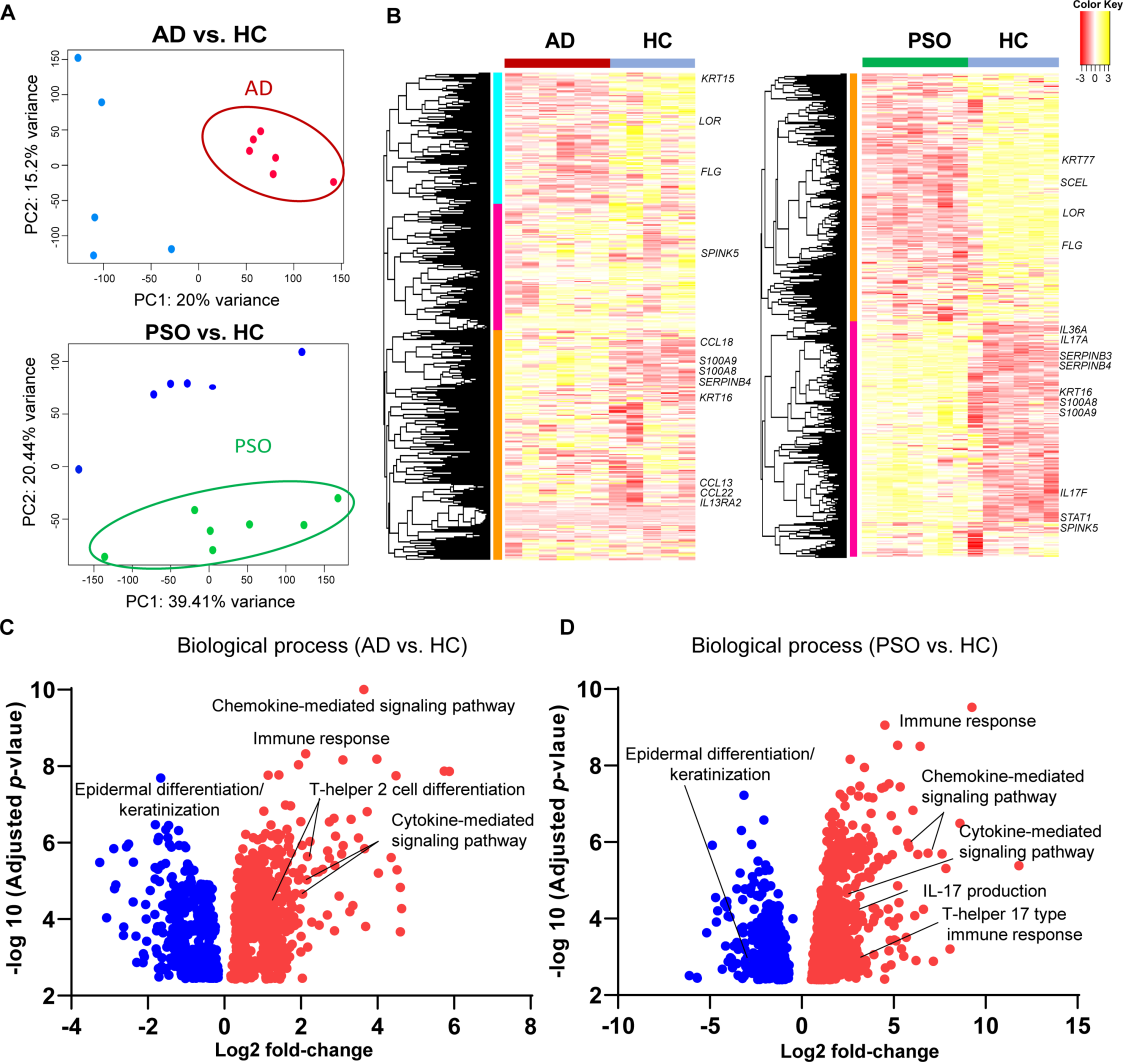
Distinct skin transcription profiles in AD, psoriasis, and healthy controls. **(A)** Principal component analysis comparing AD (up) and psoriasis (down) to HCs. Each point represents a sample, colored by group. **(B)** DEG analysis comparing each disease group to healthy controls. **(C,D)** Gene ontology biological process enrichment analysis comparing AD and psoriasis to HCs. The *x*-axis represents the log₂ fold-change in enrichment between HCs and AD group **(C)**, HCs and psoriasis group **(D)**, and the *y*-axis represents the adjusted *p*-value. Red dots indicate significantly upregulated pathways, while blue dots indicate significantly downregulated pathways. HC, healthy control; AD, atopic dermatitis; PSO, psoriasis.

First, Principal component analysis (PCA) was performed to examine the clustering patterns of AD and psoriasis relative to HCs. The analysis revealed that the AD samples were primarily separated along PC1, whereas the psoriasis samples were distinguished along PC2 (Figure 1A). These findings suggest that AD and psoriasis exhibit distinct transcriptomic profiles that reflect disease-specific immunological and pathological mechanisms. Differentially expressed gene (DEG) analysis identified 6,445 DEGs between AD group and HCs, as well as between the psoriasis group and HCs (Figure 1B). The DEG heatmap highlights the transcriptomic heterogeneity of AD, as AD samples were distributed across multiple clusters without clear separation from HCs. In contrast, psoriatic samples formed a distinct cluster, well separated from HCs, indicating a more uniform gene expression profile in psoriasis.

To further investigate the functional differences between AD and psoriasis, Gene ontology (GO) biological process enrichment analysis was performed on the upregulated and downregulated genes in each condition. The analysis revealed that both AD and psoriasis showed enrichment of immune responses and cytokine/chemokine-mediated signaling among the upregulated genes, whereas keratinization/epidermal differentiation was commonly downregulated (Figures 1C,D).

However, distinct immunoregulatory patterns have been observed. AD was associated with GO terms, such as T-helper 2 cell differentiation (Figure 1C), whereas psoriasis showed significant enrichment of T-helper 17 immune response and interleukin (IL)-17 production (Figure 1D). These results indicate that although AD and psoriasis share overarching inflammatory and skin barrier impairment pathways, they are driven by differential T cell–mediated immune mechanisms.

### 3.2 AD skin is strongly associated with Th2-mediated cytokines and chemokines

Biological process enrichment analysis revealed significant upregulation of the Th2 cell differentiation pathway in AD compared to psoriasis and HC skin, suggesting a predominantly Th2-driven immune response in AD. To further investigate this immune profile and assess its consistency with previous findings, the expression levels of key Th2-associated cytokines and chemokines were analyzed across the three groups.

**Figure 2:**
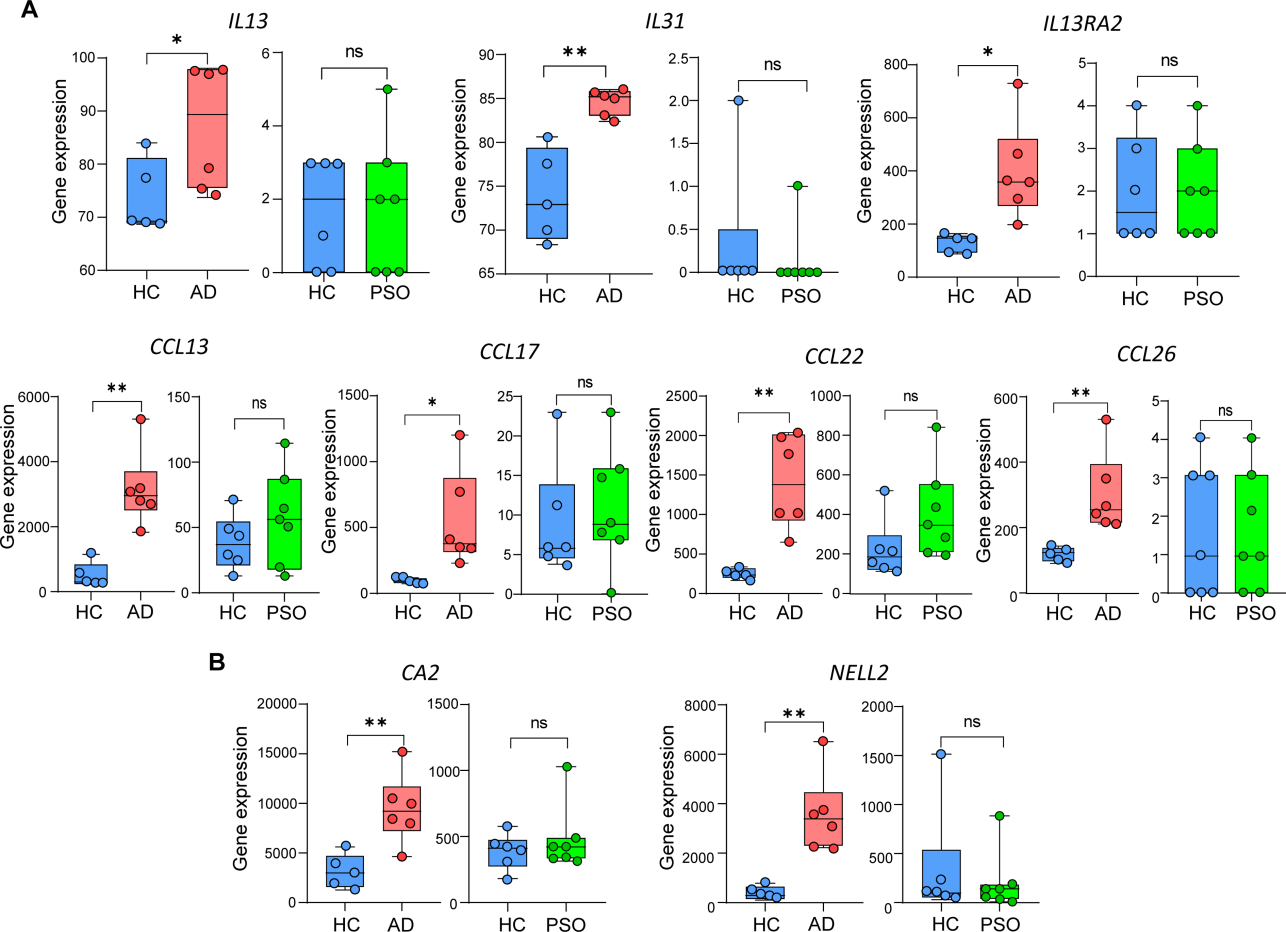
Expression analysis of Th2-related genes upregulated in AD but not differentially expressed in psoriasis compared to HCs. **(A)** Expression level of Th2-related genes (*IL13, IL31, IL13RA2, CCL13, CCL17, CCL22*, and *CCL26*) compared between HCs and AD, and between HCs and psoriasis. **(B)** Expression level of *CA2* and *NELL2,* which are induced by Th2 cytokines. Each boxplot represents differential expression between the two groups, with values displayed from minimum to maximum. Individual data points are shown, and statistical significance was determined using a two-sided Mann–Whitney U test. *p < 0.05, **p < 0.01, ns; not significant (*p* > 0.05). HC, healthy control; AD, atopic dermatitis; PSO, psoriasis.

Levels of *IL13, IL31,* interleukin 13 receptor subunit alpha-2 *(IL13RA2),* C-C motif chemokine ligand (*CCL)13, CCL17, CCL22*, and *CCL26*, previously identified as severity biomarkers in AD (19), were significantly upregulated in AD skin compared to those in the HC group, whereas their expression in psoriatic skin did not differ significantly from those in HCs (Figure 2A). In addition, carbonic anhydrase II (*CA2*) and nel-like protein 2 (*NELL2*), previously identified as AD-specific diagnostic markers induced by Th2 cytokines (19), were significantly increased in AD compared to HCs, whereas their expression levels in psoriasis were comparable to those in HCs (Figure 2B). These results are consistent with the well-established role of Th2-mediated inflammation in AD pathogenesis and reinforce the concept that AD is characterized by heightened Th2 immune activation.

Furthermore, a subset of Th2-associated genes either showed no significant differences in expression compared to HCs in both AD and psoriasis, or were similarly differentially expressed in both AD and psoriasis (Supplementary Figure S1). Although these genes have been previously implicated in Th2-driven inflammation, they may not play a dominant role in the skin pathogenesis of AD in this cohort.

### 3.3 Psoriasis skin is strongly associated with Th17-mediated cytokines and chemokines

**Figure 3:**
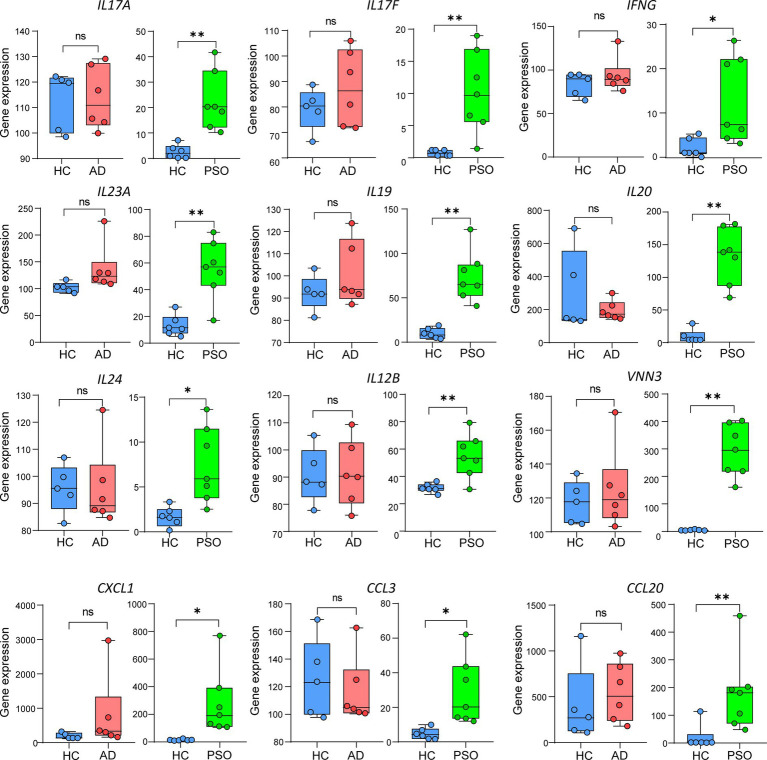
Expression analysis of Th17-related genes upregulated in psoriasis but not differentially expressed in AD compared to HCs. Expression levels of Th17-related genes (*IL17A, IL17F, IFNG, IL23A, IL19, IL20, IL24, IL12B, VNN3, CXCL1, CCL3,* and *CCL20*) compared between HCs and psoriasis, and between HCs and AD. Each boxplot represents differential expression between the two groups, with values displayed from minimum to maximum. Individual data points are shown, and statistical significance was determined using a two-sided Mann–Whitney U test. *p < 0.05, **p < 0.01, ns; not significant (*p* > 0.05). HC, healthy control; AD, atopic dermatitis; PSO, psoriasis.

Biological process enrichment analysis revealed significant upregulation of the IL-17 signaling pathway in psoriasis, suggesting a predominant Th17-driven immune response. To further investigate this immune profile and assess whether our data aligned with previous findings, the expression levels of key Th17-associated cytokines and chemokines were analyzed in psoriatic, AD, and HC skin samples. Levels of *IL17A, IL17F, IFNG, IL23A, IL19, IL20, IL24, IL12B,* vanin-3 *(VNN3),* C-X-C motif chemokine ligand (*CXCL*)*1, CCL3,* and *CCL20*, well-known psoriasis key biomarker, significantly upregulated in psoriatic skin compared with HC skin. In contrast, no significant differences in the expression of these genes were observed between AD and healthy control skin, suggesting that Th17-mediated inflammation is a distinct feature of psoriasis rather than AD (Figure 3). These findings are consistent with the well-established role of Th17-driven immune responses in the pathogenesis of psoriasis, further confirming that psoriasis is characterized by enhanced Th17 immune activation. Additionally, a subset of Th17-associated genes showed upregulation in both AD and psoriasis or exhibited no significant differences compared to HCs (Supplementary Figure S2).

### 3.4 Genes known to be involved in skin differentiation were differentially expressed between the disease and healthy control groups

**Figure 4:**
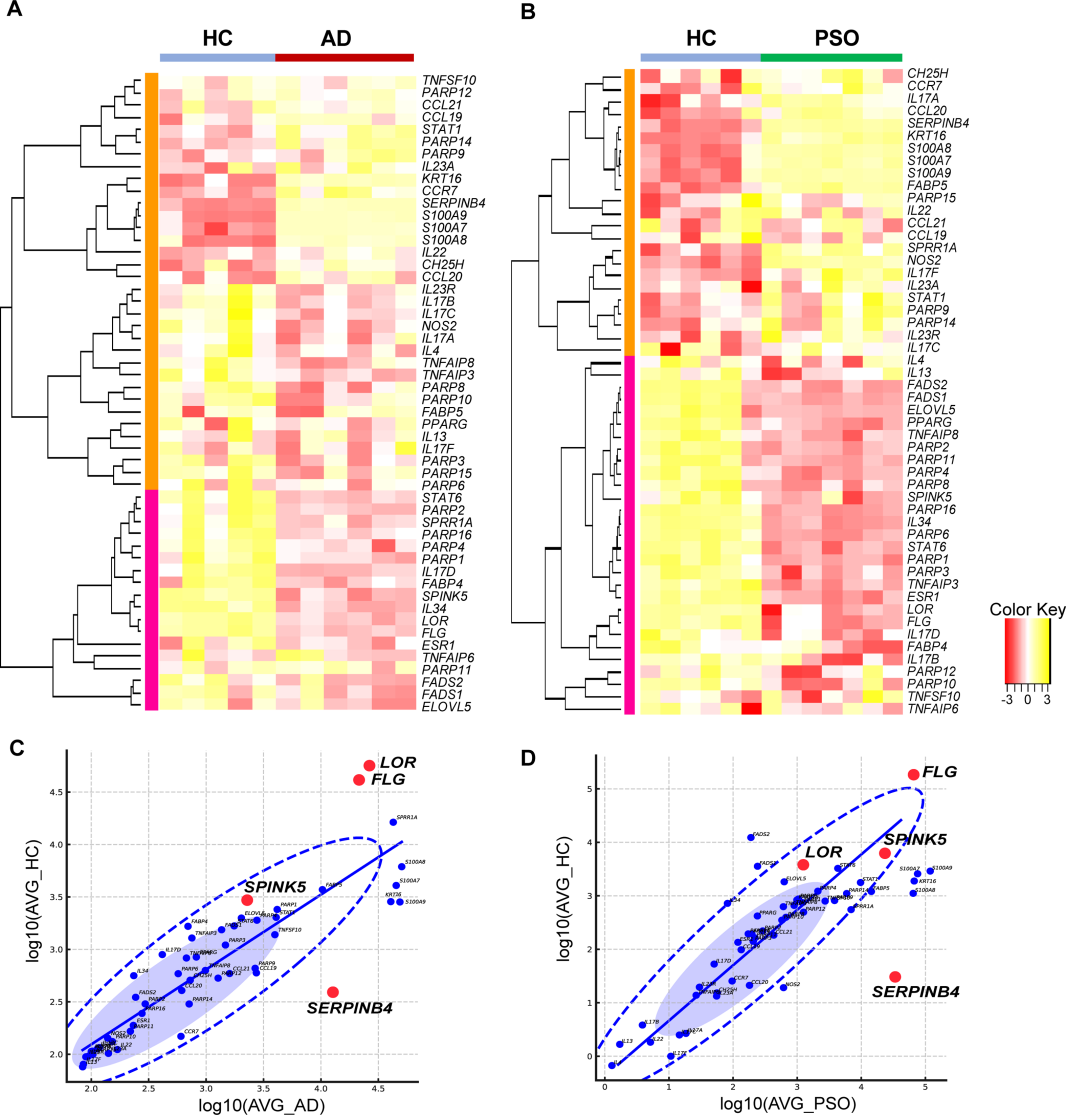
Differential expression of 52 selected genes between disease and healthy control groups. **(A)** Clustering heatmap showing gene expression patterns between the AD and HC groups based on 52 selected genes, identifying two distinct clusters. **(B)** Clustering heatmap showing gene expression patterns between the psoriasis and HC groups based on 52 selected genes and identifying two clusters. **(C,D)** Scatterplot of the standardized mean gene expression levels, comparing AD and psoriasis to HC group. HC, healthy control; AD, atopic dermatitis; PSO, psoriasis.

Among the 6,445 DEGs, genes meeting the criteria of adjusted *p*-value<0.05 and |log₂ fold change| > 2 were initially identified. From these, 52 genes were further selected based on prior literature indicating their relevance to cutaneous inflammatory responses (20–25). Subsequent DEG analysis was conducted on the 52 selected genes to evaluate the differences among the AD, psoriasis, and healthy control groups (Figures 4A,B). Scatterplot analysis identified four key genes *FLG, LOR, SPINK5,* and *SERPINB4* that exhibited significant differences between the disease and healthy control groups, or had strong known associations with AD and psoriasis. Among these, *FLG* and *SERPINB4* demonstrated pronounced differential expression between both AD and psoriasis groups compared to HCs, while *LOR* also showed marked differential expression, particularly prominent in AD and significantly reduced in psoriasis. Notably, *SPINK5* exhibited disease-specific expression patterns, characterized by higher expression in HCs compared to AD, yet increased expression in psoriasis relative to HCs, highlighting its potential role as a distinguishing biomarker between the two diseases (Figures 4C,D).

Although *S100A7, S100A8, S100A9*, and keratin 16 (*KRT16)* displayed substantial differences in expression levels in the same scatterplot analysis, these genes consistently showed elevated expression in both AD and psoriasis groups (Supplementary Figure S3), limiting their ability to discriminate between the two diseases. Therefore, these genes were not included among the final set of key genes.

Collectively, these findings suggest that skin differentiation-related genes play crucial roles in the pathogenesis of AD and psoriasis.

### 3.5 Differential expression of skin barrier-related genes in AD and psoriasis

**Figure 5:**
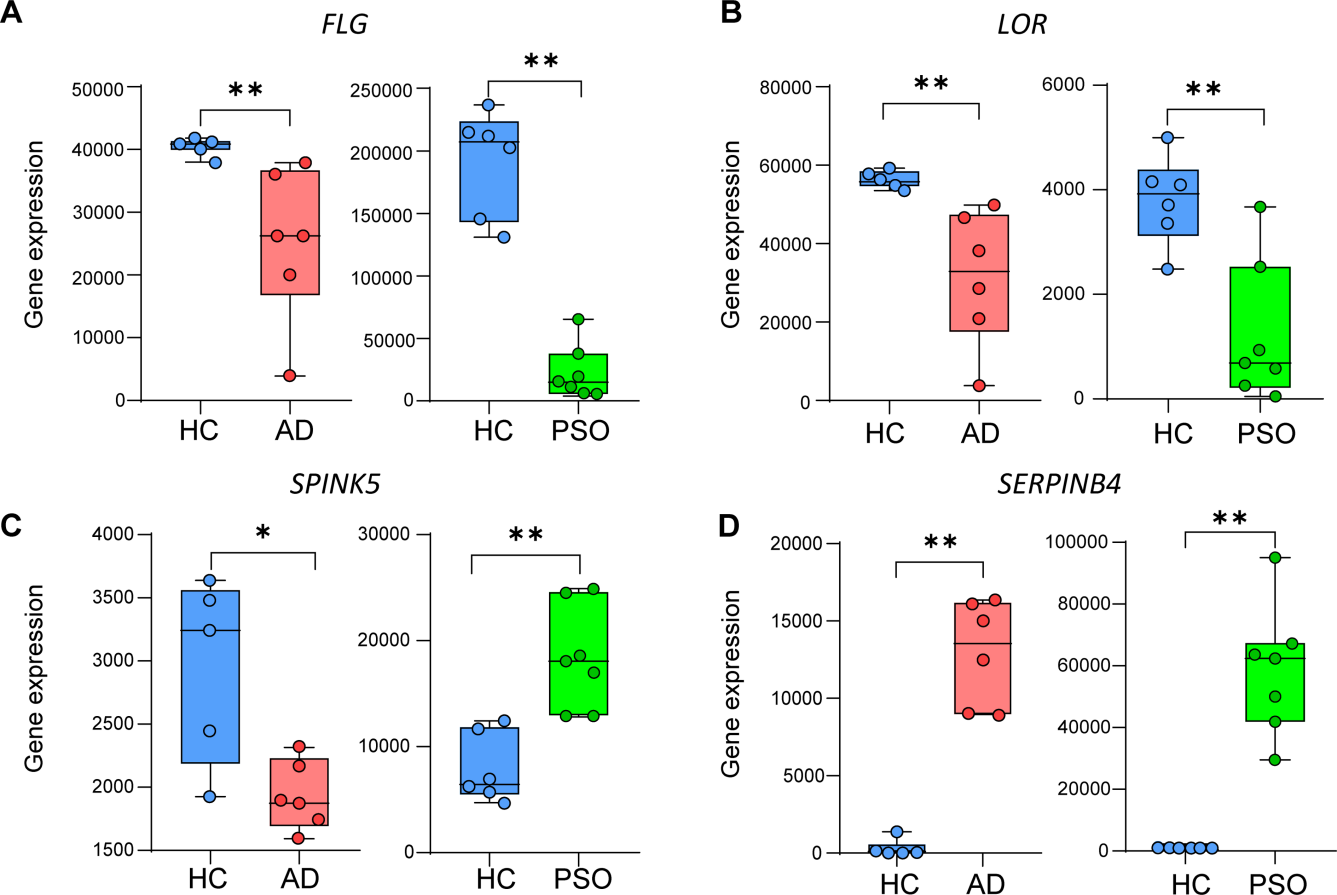
Boxplot analysis of *FLG*, *LOR*, *SPINK5*, and *SERPINB4* gene expression between disease and healthy control groups. Expression levels of **(A)** *FLG*, **(B)** *LOR*, **(C)** *SPINK5*, and **(D)** *SERPINB4* were compared between HCs and AD and between HCs and psoriasis. Each boxplot represents differential expression between the two groups, with values displayed from minimum to maximum. Individual data points are shown, and statistical significance was determined using a two-sided Mann–Whitney U test. *p < 0.05, **p < 0.01. HC, healthy control; AD, atopic dermatitis; PSO, psoriasis.

*FLG*, which encodes filaggrin, plays crucial roles in skin barrier formation and keratinocyte differentiation. *FLG* expression was significantly decreased in both AD (*p* = 0.0043, 95% confidence interval [CI]) and psoriasis (*p* = 0.0012, 95% CI) patients compared to that in the healthy control group (Figure 5A).

*LOR*, which encodes loricrin, is essential for epidermal differentiation and skin barrier integrity. *LOR* expression was significantly reduced in the AD group (*p* = 0.0043, 95% CI) compared to that in the healthy control group. *LOR* expression was also decreased in patients with psoriasis (*p* = 0.0082, 95% CI; Figure 5B).

*SPINK5*, which is associated with allergic diseases such as AD and asthma and is known to influence IgE regulation, exhibited significantly higher expression in patients with psoriasis than in healthy controls (*p* = 0.0012, 95% CI). In contrast, *SPINK5* expression in patients with AD was downregulated compared to healthy controls (*p* = 0.0173, 95% CI; Figure 5C).

*SERPINB4*, which is involved in epidermal barrier homeostasis and has been implicated in chronic inflammatory skin diseases, was significantly upregulated in both AD (*p* = 0.0043, 95% CI) and psoriasis (*p* = 0.0012, 95% CI) patients compared to healthy controls (Figure 5D).

To validate our transcriptomic findings, we first performed cross-platform normalization between microarray (HC vs. AD) and RNA-seq (HC vs. psoriasis) datasets. PCA before and after normalization showed that platform-associated separation was reduced after Rank-In normalization, as indicated by the decreased platform-based silhouette score (Supplementary Figure S4A). The expression patterns of the *FLG*, *LOR*, *SPINK5*, and *SERPINB4* genes remained consistent after normalization (Supplementary Figures S4B,C), confirming that our results were not driven by platform heterogeneity. Furthermore, quantitative reverse transcription PCR (RT-qPCR) analysis using skin tissues from HC, AD and psoriasis patients revealed expression changes consistent with our transcriptomic results (Supplementary Figure S5). Direct statistical comparisons between AD and psoriasis showed that, among the four genes, *SPINK5* was the only gene with a statistically significant difference at both the transcriptomic and mRNA validation levels (Supplementary Figures S4C, S5). In contrast, FLG, LOR, and SERPINB4, while showing concordant directional changes in both diseases, did not reach statistical significance in direct AD-versus-psoriasis comparisons, suggesting that these genes reflect shared barrier dysfunction rather than disease-specific differences.

These findings indicate dysregulated expression of key skin barrier-associated genes in both AD and psoriasis, suggesting overlapping yet partially distinct alterations in epidermal differentiation and immune-related pathways in these diseases.

### 3.6 Validation of FLG, LOR, LEKTI, and SERPINB4 expression in human skin samples

To validate the transcriptomic analysis results at the protein level, immunofluorescence staining was performed on AD, psoriasis, and healthy skin samples to assess the expression of FLG, LOR, the *SPINK5*-encoded protein lymphoepithelial Kazal-type inhibitor (LEKTI), and SERPINB4. Immunofluorescence and quantitative imaging were performed to compare the expression patterns of these proteins.

**Figure 6:**
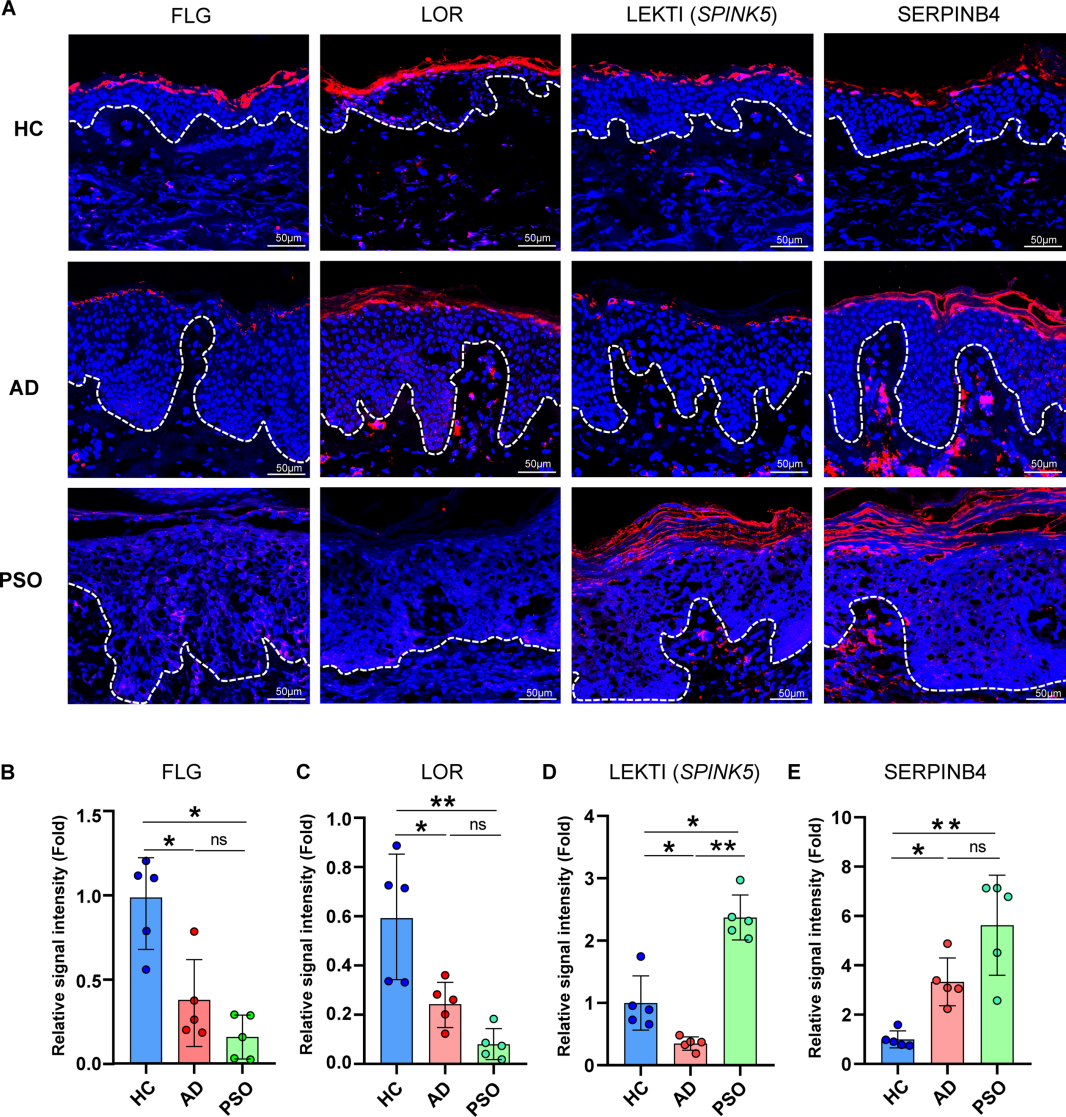
Immunofluorescence analysis of human skin samples from patients with AD, psoriasis, and HCs. **(A)** Representative immunofluorescence images of human AD, psoriasis, and healthy skin. Red staining depicts FLG, LOR, LEKTI, or SERPINB4, as indicated. Nuclear counterstaining is shown in blue. Dashed lines represent the epidermal/dermal boundary. Scale bars: 50 μm. **(B–E)** Relative signal intensity of FLG, LOR, LEKTI, and SERPINB4 expression was measured using immunofluorescence staining. Fluorescence intensity was quantified as integrated density values using ImageJ and normalized to the mean intensity of the healthy skin group. Data are presented as mean ± *SD*. Individual data points are shown, and statistical significance was determined using the Kruskal–Wallis test followed by Dunn’s multiple comparisons test. *p < 0.05, **p < 0.01, ns; Not significant (*p* > 0.05). HC, healthy control; AD, atopic dermatitis; PSO, psoriasis.

The analysis revealed that FLG and LOR proteins were strongly expressed in the granular epidermal layer of healthy skin but were markedly reduced in both AD and psoriatic skin (Figures 6A–C). This trend was consistent with the gene expression patterns observed in the transcriptomic analysis. LEKTI was also expressed in the granular layer of healthy skin, with increased expression in psoriatic skin compared to that in healthy controls, whereas its expression tended to decrease in AD skin (Figures 6A,D). In contrast, SERPINB4 was expressed in both the spinous and granular layers of healthy skin and was significantly upregulated in both AD and psoriatic skin (Figures 6A,E). Direct comparison between AD and psoriasis revealed that SPINK5/LEKTI showed consistent and statistically significant differential expression between the two diseases, further supporting its disease-specific expression pattern. Collectively, these findings confirm that dysregulated expression of FLG, LOR, SPINK5/LEKTI, and SERPINB4 occurs at both the transcriptomic and protein levels, further supporting their potential roles in the pathophysiology of AD and psoriasis.

## 4 Discussion

In this study, we aimed to elucidate the molecular mechanisms underlying barrier dysfunction and immune dysregulation in AD and psoriasis by examining the expression of key genes involved in epidermal integrity and inflammation. The expression levels of *FLG*, *LOR*, *SPINK5*, and *SERPINB4* was markedly altered in both diseases compared to HCs.

*FLG* is a large histidine-rich protein that aggregates into keratin intermediate filaments (26). This process is important for the structure and function of the stratum corneum. The stratum corneum provides a physical barrier and first-line host defense against the environment and pathogens (27). Loss- of-function mutations in the *FLG* gene are a well-established risk factor for AD (28–30), with a meta-analysis reported odds ratios ranging from 3.12 to 4.78 (31). Although AD is a polygenic disease, *FLG* mutations are particularly prevalent in Caucasian populations. Beyond genetic alterations, *FLG* expression is also suppressed by Th2 cytokines, such as IL-4 and IL-13, through the JAK/STAT pathway, particularly via STAT3 (32, 33). In contrast, while *FLG* gene expression was significantly lower in psoriatic skin than in normal skin in our study, the role of *FLG* mutations in psoriasis remains controversial. Some studies in Caucasians report no associations (34), while others in Chinese and Korean populations suggest potential relevance (35). In the present study of Asian patients with AD, *FLG* was therefore considered a key gene not primarily because of its genetic variant frequency, but because its consistent downregulation in lesional skin reflects a functional impairment of the epidermal barrier that is directly relevant to disease activity.

*LOR* encodes the loricrin protein, a major structural protein expressed in the granular layer of keratinized epithelial cells. It plays a crucial role in epidermal differentiation and skin barrier formation and is one of the key genes within the epidermal differentiation complex (32, 36). In AD, *LOR* downregulation occurs through both genetic susceptibility and the suppressive action of IL-4 and IL-13 via the STAT6 pathway (36). In psoriatic skin, *LOR* expression is also diminished. However, this is not driven by Th2 cytokines, which are not elevated in psoriasis. Instead, proinflammatory cytokines such as IL-17A, IL-22, and TNF-alpha activate NF-κB and JNK signaling pathways in keratinocytes, leading to impaired differentiation and barrier dysfunction (23).

Taken together, deficiencies of *FLG* and *LOR* contribute to disease pathogenesis in AD and psoriasis through distinct mechanisms. In AD, these deficiencies originate from both genetic and Th2 cytokine-mediated transcriptional repression, resulting in barrier impairment that promotes microbial colonization and allergen entry, thereby exacerbating Th2-driven inflammation and chronicity. In psoriasis, the reduction of *FLG* and *LOR* is not a primary cause but rather a secondary effect of cytokine-driven inflammation that disrupts epidermal differentiation, reinforces barrier dysfunction, and sustains the chronic inflammatory loop characteristic of the psoriatic phenotype.

*SPINK5* encodes LEKTI, which functions as a 15-domain serine proteinase inhibitor (37–39). There are 34 mutations that have been identified in the *SPINK5* gene in Netherton syndrome patients, which introduce premature termination codons that result in truncated, nonfunctional proteins. *SPINK5* mutations may cause excessive kallikrein (KLK)5/7 activity, which degrades lipid-processing enzymes and corneodesmosome constituent proteins, resulting in defects in the permeability barrier (40). This culminates in defective barrier function and promotes Th2-skewed inflammation via protease-activated receptor 2 (PAR2)-mediated thymic stromal lymphopoietin (TSLP) induction, mirroring the pathophysiology observed in AD (41, 42). In our study, SPINK5 expression was lower in AD skin compared to HCs. Several studies in Japan have also reported associations between *SPINK5* polymorphisms and AD (43, 44), and Kabesch et al. further demonstrated its link to asthma in children with AD, suggesting a broader role for serine protease inhibitors in both cutaneous and airway allergic diseases (45). These findings indicate that *SPINK5* polymorphisms may be particularly relevant to allergic disorders in Asian populations.

Notably, *SPINK5* expression was increased in psoriatic skin, in contrast to the uniform downregulation of *FLG* and *LOR* observed across both AD and psoriasis. Previous studies have shown that TNF-*α* and IL-17A can upregulate *SPINK5* expression in keratinocytes (46). Our findings support this, as IL-17A levels were significantly higher in psoriatic skin compared to HCs. In psoriasis, increased *SPINK5* expression may serve as a compensatory mechanism to regulate heightened protease activity, particularly KLK6, KLK10, KLK11, and KLK13, thereby supporting barrier integrity and preventing excessive skin shedding (46). In contrast to AD, where *SPINK5* deficiency contributes to pathogenesis, psoriasis appears to exhibit compensatory *SPINK5* upregulation in response to inflammation-induced protease activity. These findings suggest that the role of *SPINK5* differs between disease states, with genetic variants contributing to AD susceptibility, particularly in Asian populations, and inflammatory stimuli enhancing *SPINK5* expression in psoriasis as a compensatory response.

Finally, we examined the expression levels of *SERPINB4* in the skin of patients with AD and psoriasis compared to HCs. In AD, *SERPINB4* is induced by Th2 cytokines such as IL-4 and IL-13, leading to the upregulation of proinflammatory mediators including *S100A8*. This promotes early inflammation, impairs skin barrier function, and facilitates immune cell infiltration, thereby amplifying Th2-skewed immune responses (47, 48). In psoriasis, *SERPINB4* expression is upregulated by Th17-related cytokines such as IL-17A and TNF-*α*, which activate the NF-κB pathway in keratinocytes and induce the expression of inflammatory chemokines such as CXCL1, CXCL2, and CCL20 (49, 50). Notably, Pso p27, derived from *SERPINB4*, is specifically detected in psoriatic lesions and its expression correlates with disease severity and chronicity (51, 52). Moreover, studies have shown that *SERPINB3/B4* deficiency suppresses NF-κB-mediated inflammation, indicating its role in chronic immune activation (49). Thus, *SERPINB4* plays a pathogenic role in both AD and psoriasis via divergent immune pathways, being Th2-associated in AD and Th17/NF-κB-driven in psoriasis.

When compared with HCs, *SPINK5* expression was decreased in AD but increased in psoriasis, highlighting its potential as a preliminary candidate molecule showing a distinct expression pattern between the two diseases. In contrast, *FLG* and *LOR* were consistently downregulated, and *SERPINB4* was upregulated in both conditions, reflecting shared pathogenic pathways related to barrier dysfunction and chronic inflammation. To assess clinical relevance, we examined correlations between gene expression and disease severity scores. *SERPINB4* expression showed significant associations with severity in both AD and psoriasis, while *SPINK5* was significantly correlated with psoriasis severity and showed an inverse trend in AD. *FLG* and *LOR* also displayed inverse but non-significant correlations. These findings indicate that *SPINK5* may serve as a preliminary molecular indicator reflecting differential expression patterns between AD and psoriasis, whereas *FLG*, *LOR*, and *SERPINB4* mainly reflect common pathogenic mechanisms underlying both diseases (Supplementary Figure S6).

This study has several limitations. First, the number of samples analyzed was relatively small. As a result, the findings may not be fully generalizable to the broader Asian population with AD and psoriasis. Second, although we discussed the potential biological implications of the observed gene expression changes, the proposed mechanisms are based primarily on transcriptomic analysis and interpretation of existing literature. Additional functional experiments and mechanistic studies will be necessary to validate the roles of these genes in disease pathogenesis. Third, different transcriptomic platforms were used with microarray for AD and RNA-seq for psoriasis. While this may introduce technical bias, we attempted to minimize and assess platform-related effects by analyzing each disease group in comparison with its own HCs and by performing Rank-In based cross-platform normalization. Nevertheless, cross-disease comparisons should be interpreted cautiously because residual platform-related effects may remain. Fourth, transcriptomic profiling and RT-qPCR were performed using the same RNA samples, which allowed direct validation of selected gene expression patterns at the transcript level within the same small cohort. However, immunofluorescence validation was performed in a separate cohort with similarly limited sample size. This small sample size and separate cohort design may limit the robustness of the results and the generalizability of our conclusions. Finally, because a larger independent validation cohort was not included, the diagnostic utility and therapeutic relevance of the selected genes should be interpreted as preliminary and require further validation.

Further studies should include functional experiments to validate the biological relevance of the four key genes expression changes. *In vitro* assays using patient-derived keratinocytes could be stimulated with Th2- or Th17-associated cytokines to directly assess the regulation of *FLG*, *LOR*, *SPINK5*, and *SERPINB4* expression under disease-relevant immune conditions. In addition, functional assays targeting *SPINK5* and *SERPINB4*, as well as *in vivo* validation in AD and psoriasis mouse models, will be essential to determine their potential contributions to barrier dysfunction and inflammation.

**Figure 7:**
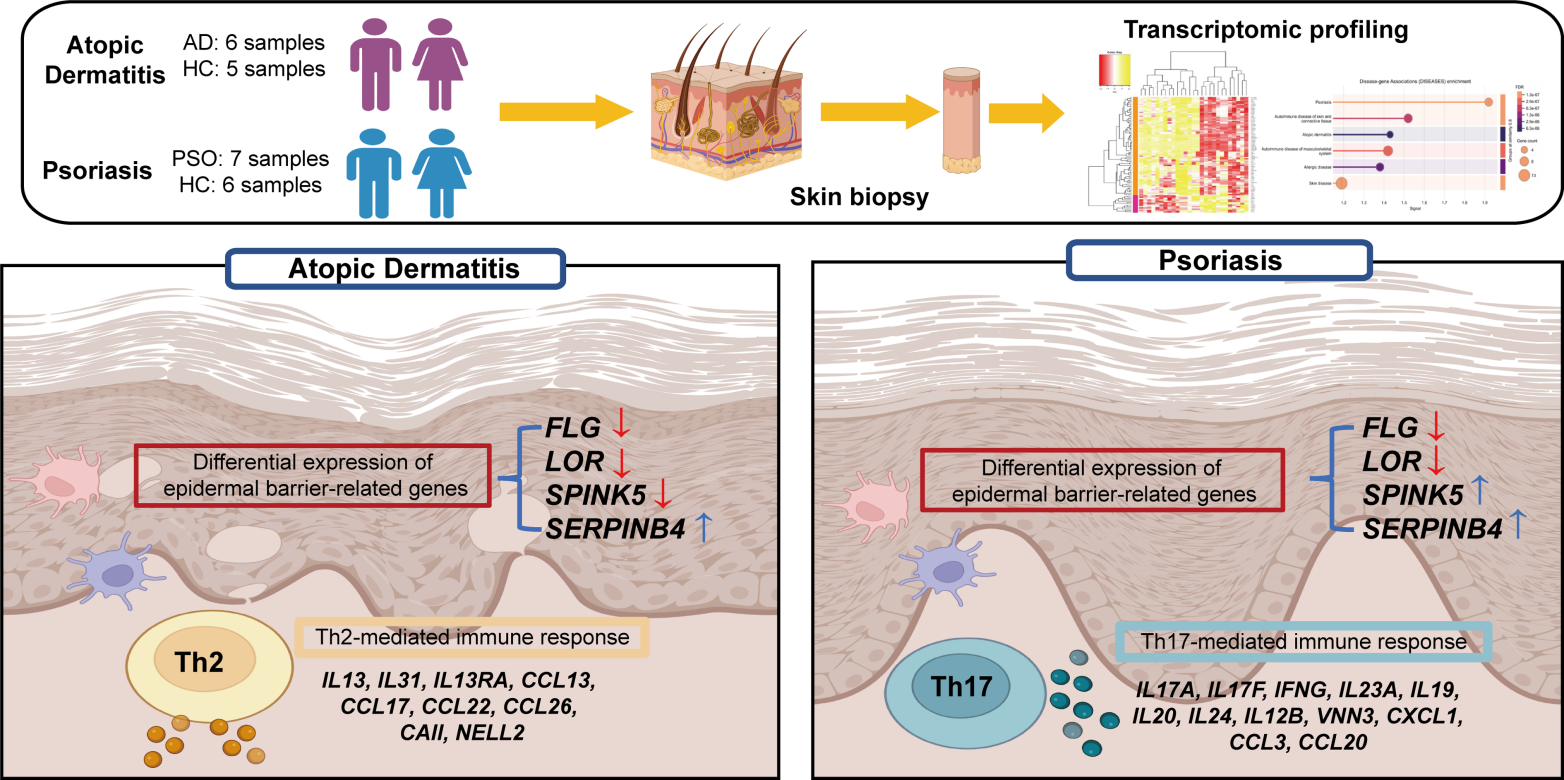
A schematic overview of the AD and psoriasis gene expression profiles. AD is associated with a Th2-dominant immune response, while psoriasis is characterized by a Th17-dominant immune response. The figure also illustrates the differential expression of *FLG, LOR, SPINK5*, and *SERPINB4* in AD and psoriasis, as identified in the transcriptomic analysis. AD, atopic dermatitis; PSO, psoriasis; HC, healthy control; *FLG*, filaggrin; *LOR*, loricrin; *SPINK5*, Serine protease inhibitor Kazal-type 5; *SERPINB4*, Serpin family B member 4. Created in BioRender. Park (2026). https://BioRender.com/xxsy7qv.

Overall, our findings provide valuable insights into the pathophysiology of inflammatory skin diseases in the Asian population. Specifically, the dysregulation of barrier-related genes (*FLG, LOR, SPINK5*) may contribute to impaired epidermal integrity, enhanced antigen penetration, and aberrant immune activation, promoting Th2-skewed inflammation in AD and Th17-dominant responses in psoriasis. Elevated *SERPINB4* expression may further amplify chronic inflammation in both diseases (Figure 7). Notably, *FLG*, *LOR*, and *SERPINB4* showed shared expression alterations in both diseases, whereas *SPINK5* demonstrated the most consistent differential expression pattern between AD and psoriasis, suggesting its potential as a candidate molecular signature for disease discrimination. While these gene expression patterns are generally consistent with previous reports from Western and Asian cohorts, the present study emphasizes a comparative interpretation between AD and psoriasis, allowing shared and disease-specific molecular features to be more clearly delineated. These findings highlight the interplay between barrier dysfunction and immune dysregulation and may inform future research on the molecular pathogenesis of these diseases.

## Data Availability

Microarray datasets are available through the National Center for Biotechnology Information Gene Expression Omnibus (GEO) database under accession numbers GSE124700 (2 HC and 2 AD samples), GSE146352 (1 HC and 2 AD samples), and GSE320223 (2 HC and 2 AD samples). RNA-seq data for psoriasis and HC samples have been deposited in the GEO database under accession number GSE295540. Further information is available from the corresponding author upon reasonable request.
