# Supplementary material for: Comparative analysis of candidate gene expression in Asian atopic dermatitis and psoriasis: identification of shared and distinct molecular features
Source: Front Med (Lausanne). 2026 Aug 4;13:1878876. doi: 10.3389/fmed.2026.1878876 (PMC13481191; doi:10.3389/fmed.2026.1878876)
Supplement: Supplementary file 1 [file Data_Sheet_1.PDF]

Supplementary Material

1 Supplementary Figures and Tables

1.1 Supplementary Figures

Supplementary Figure 1

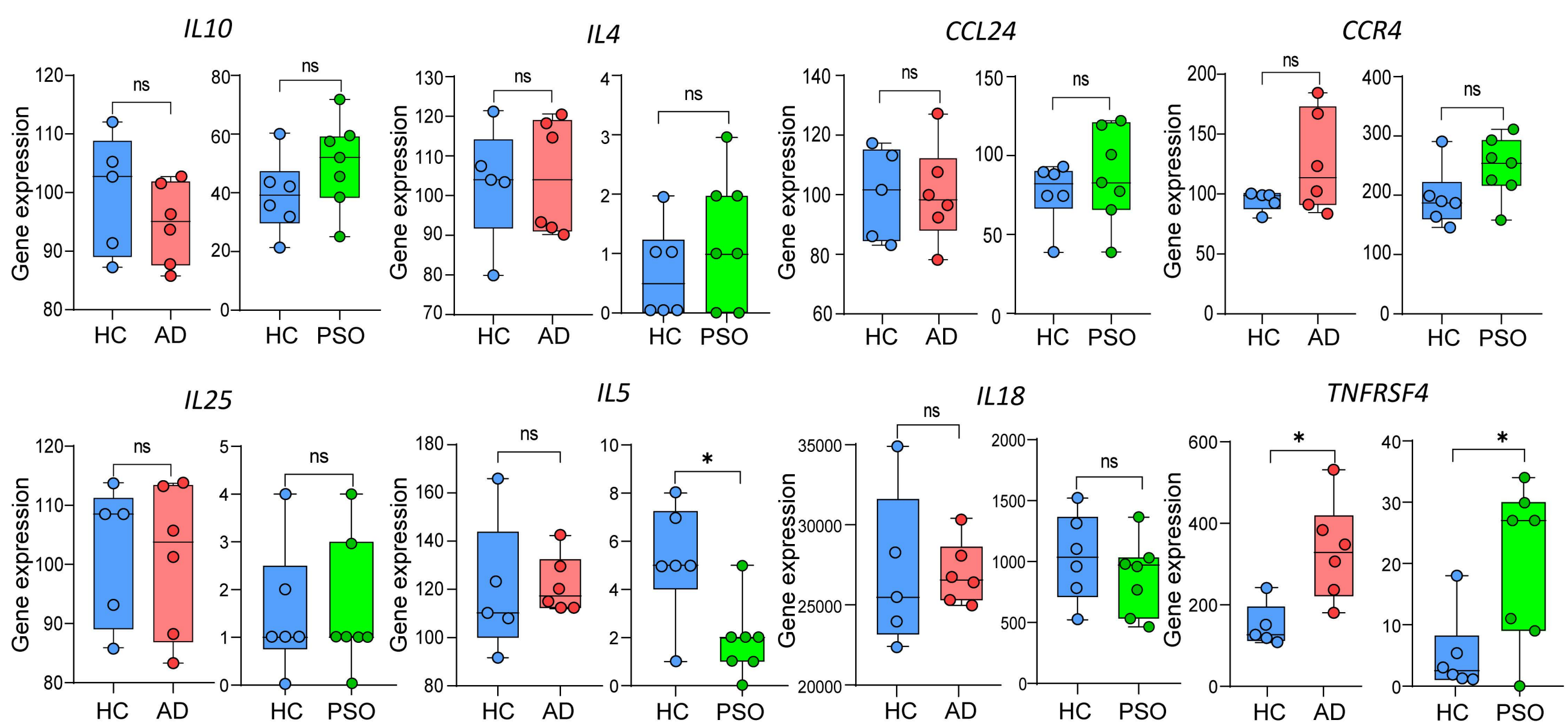

Supplementary Figure 1. Additional gene expression analysis of Th2-associated and discriminatory markers between AD and psoriasis. Expression level of Th2-associated genes showing either no significant differences between AD or psoriasis compared to HCs, or differential expression in both AD and psoriasis. Each boxplot represents differential expression between the two groups, with values displayed from minimum to maximum. Individual data points are shown, and statistical significance was determined using a two-sided Mann–Whitney U test. * $p < 0.05$, ns, not significant ($p > 0.05$). HC, healthy control; AD, atopic dermatitis; PSO, psoriasis; *TNFRSF4*, tumor necrosis factor receptor superfamily member 4

Supplementary Figure 2

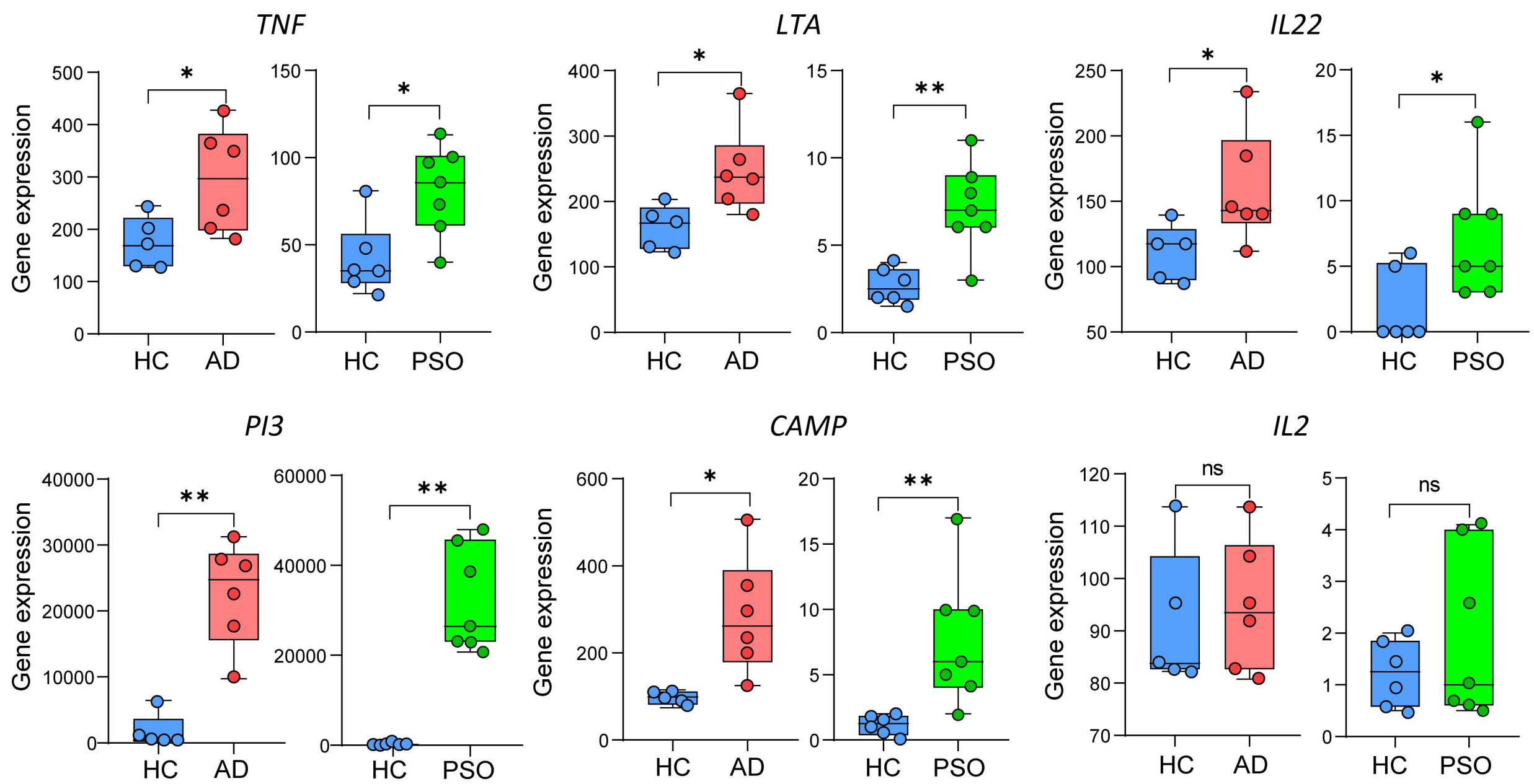

Supplementary Figure 2. Additional gene expression analysis of Th17-associated markers between AD and psoriasis. Expression level of Th17-associated genes showing either no significant differences between AD or psoriasis compared to HCs, or differential expression in both AD and psoriasis. Each boxplot represents differential expression between the two groups, with values displayed from minimum to maximum. Individual data points are shown, and statistical significance was determined using a two-sided Mann–Whitney U test. * p <0.05, ** p<0.01, ns, not significant (p >0.05). HC, healthy control; AD, atopic dermatitis; PSO, psoriasis; *TNF*, tumor necrosis factor; *LTA*, lymphotoxin alpha; *PI3,* peptidase inhibitor 3; *CAMP*, cathelicidin antimicrobial peptide.

Supplementary Figure 3

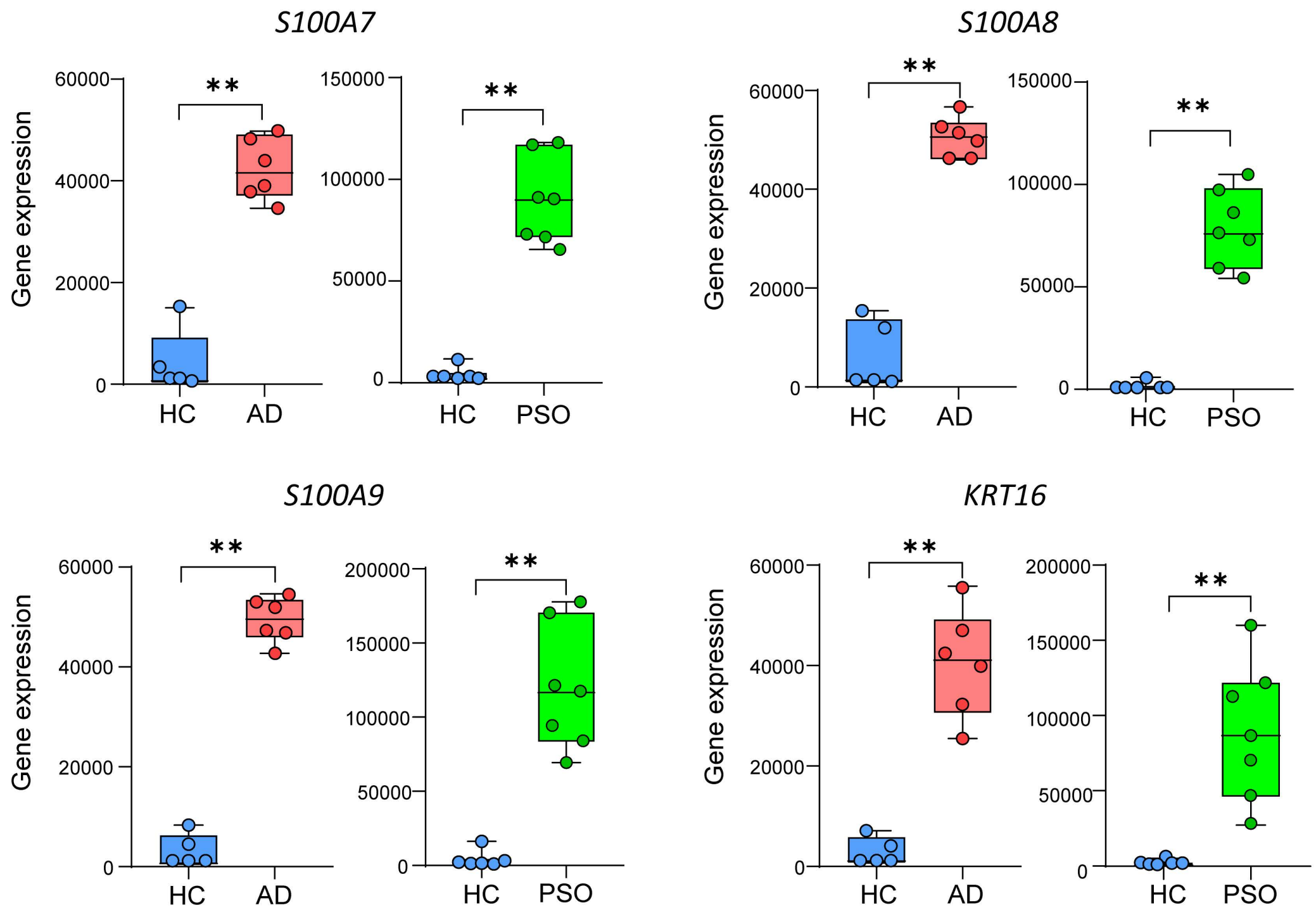

Supplementary Figure 3. Gene expression analysis of genes highly expressed in both AD and psoriasis. Expression level of Th17/Th22-associated genes (*Sl00A*s) and an epidermal hyperplasia/proliferation-related gene (*KRT16*) significantly upregulated in both AD and psoriasis compared to HCs. Each boxplot represents differential expression between the two groups, with values displayed from minimum to maximum. Individual data points are shown, and statistical significance was determined using a two-sided Mann–Whitney U test. ** p<0.01. HC, healthy control; AD, atopic dermatitis; PSO, psoriasis; *S100A7/8/9*, S100 calcium-binding protein; *KRT16*, keratin 16.

Supplementary Figure 4

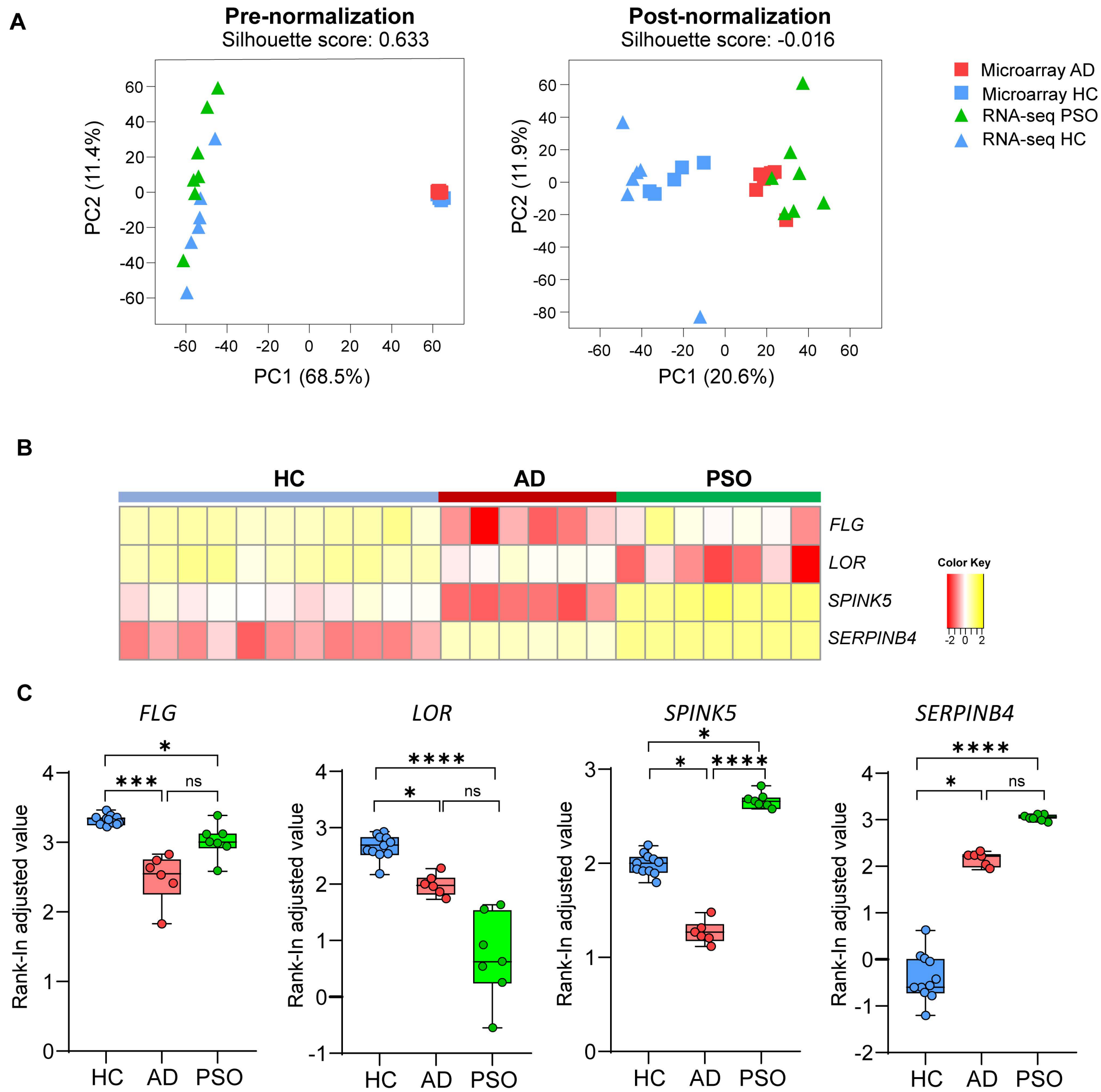

Supplementary Figure 4. Rank-In-normalized expression of four key genes in HC, AD, and psoriasis. (A) PCA plots before and after Rank-In cross platform normalization. Platform-based silhouette scores are shown in each PCA plot. Lower silhouette score indicates reduced clustering according to transcriptomic platform. (B) Heatmap visualization of *FLG, SPINK5, LOR*, and *SERPINB4* in HC, AD, and psoriasis using Rank-In–adjusted values. Expression values were standardized as gene-wise Z-scores to highlight relative differences across samples. (C) Boxplots depicting the distribution of Rank-In–adjusted values for the same four genes across HC, AD, and psoriasis. Each boxplot displays differential expression across groups, with values displayed from minimum to maximum. Individual data points are shown, and statistical significance was determined using the Kruskal–Wallis test followed by Dunn's multiple comparisons test. * $p<0.05$, ***$p< 0.001$, **** $p <0.0001$, ns, not significant ($p >0.05$). HC, healthy control; AD, atopic dermatitis; PSO, psoriasis.

Supplementary Figure 5

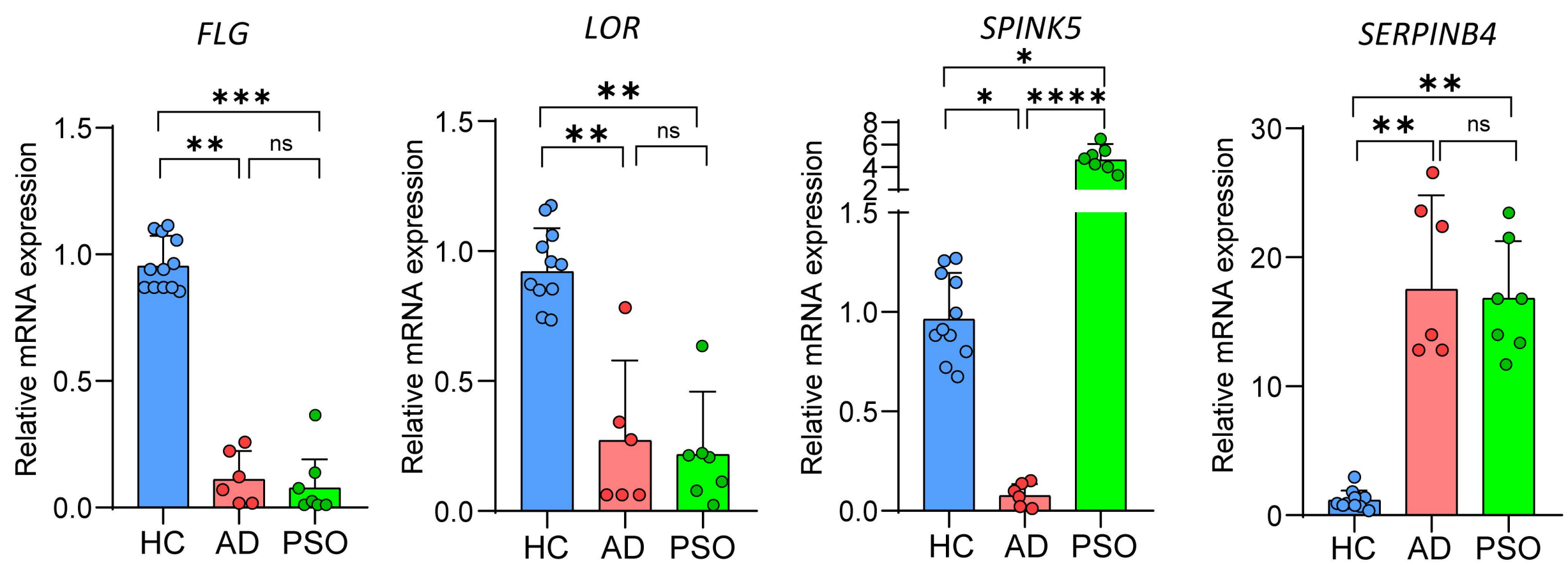

Supplementary Figure 5. Validation of differential expression of four key genes in HC, AD, and psoriasis by quantitative RT-PCR. mRNA expression levels of *FLG*, *SPINK5*, *LOR*, and *SERPINB4* were quantified in skin biopsy samples from HC, AD, and psoriasis patients. Expression values were normalized to housekeeping genes (*GAPDH*) and presented as relative expression. Data are presented as mean ± SD. Individual data points are shown, and statistical significance was determined using the Kruskal–Wallis test followed by Dunn's multiple comparisons test. * $p < 0.05$, ** $p < 0.01$, *** $p < 0.001$, **** $p < 0.0001$, ns, not significant ($p > 0.05$). HC, healthy control; AD, atopic dermatitis; PSO, psoriasis.

Supplementary Figure 6

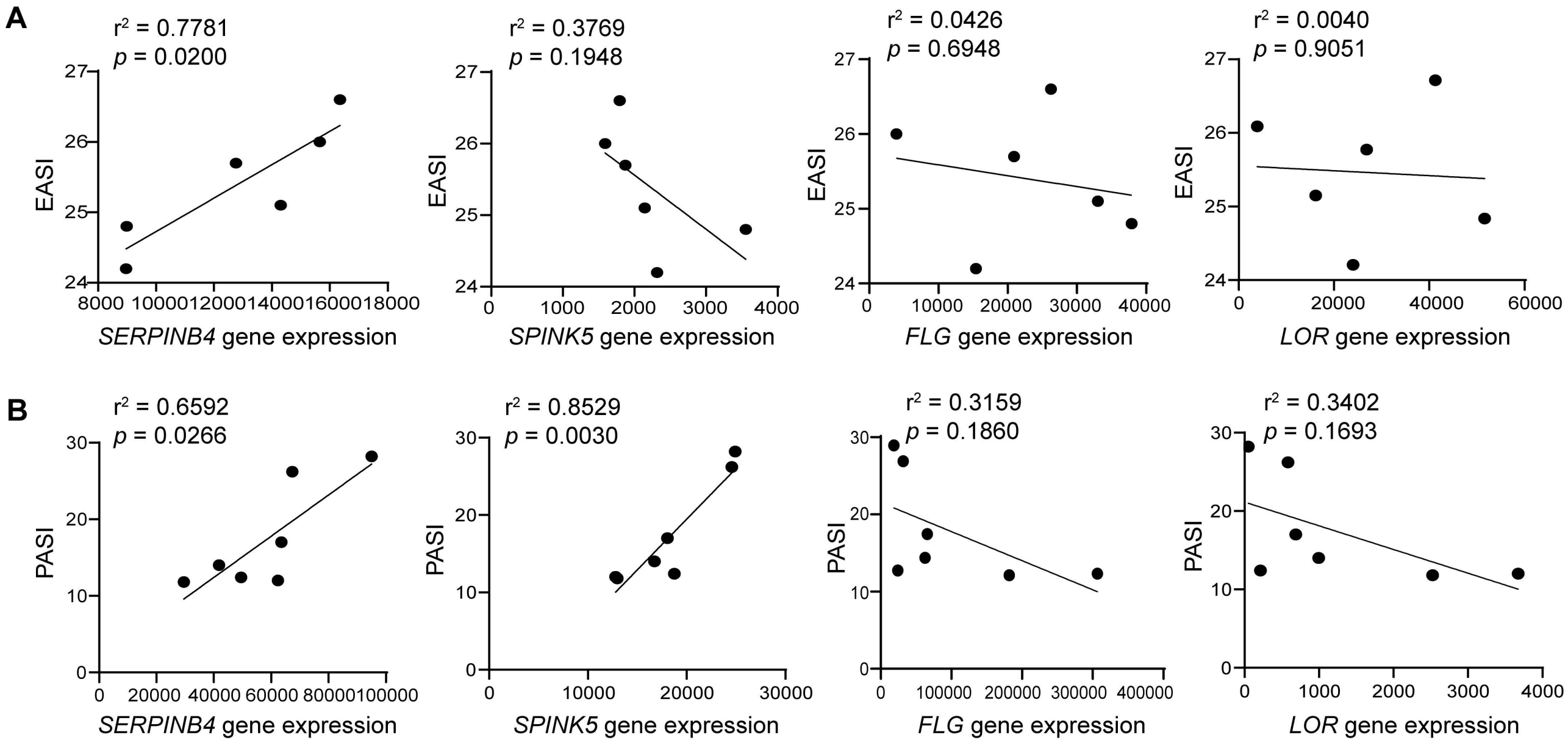

Supplementary Figure 6. Correlation between expression of four key genes and disease severity in AD and psoriasis. (A, B) Pearson's correlation coefficient analysis of *FLG*, *LOR*, *SPINK5*, and *SERPINB4* derived from transcriptomic data and disease severity scores (EASI for AD, PASI for psoriasis) was examined. Each dot represents one individual patient sample. Statistical significance was evaluated using the *p*-value obtained from the Pearson's correlation coefficient test, and the coefficient of determination (r^2) is indicated for each analysis. HC, healthy control; AD, atopic dermatitis; PSO, psoriasis; EASI, eczema area and severity index; PASI, psoriasis area and severity index.

Table S1. Clinical characteristics of patients with AD, psoriasis, and HCs

Characteristics	AD	Psoriasis	HC	*p*-value
No. subjects	11	12	16	
Age, yrs (mean ± SD)	35.25±14.64	47.86±16.29	37.4±15.55	0.3385
Sex				0.3341
Male (n)	7	7	8	
Female (n)	4	5	8	
EASI, score (mean ± SD)	25.42±0.9			
PASI, score (mean ± SD)		17.37±6.97		
Disease duration, yrs (mean ± SD)	27.01±8.51	19.75±10.85		
AD phenotype,				
Extrinsic AD, n (%)	10 (90.9%)			
Intrinsic AD, n (%)	1 (9.1%)			
Total IgE, kU/liter (mean ± SD)	3358.32±1980.13			
Eosinophil counts, /uL (mean ± SD)	1748.57±1368.5			
Psoriasis subtype, n (%)		Plaque psoriasis, 12 (100%)		

One-way ANOVA was done for statistical analysis. AD, atopic dermatitis; HC, healthy control; EASI, eczema area and severity index; PASI, psoriasis area and severity index.

Table S2. Sequences of primers used for RT-qPCR analysis

**Quantitative PCR primers**		
**Gene name**	**Forward**	**Reverse**
GAPDH	5′- ACCACAGTCCATGCCATCAC -3′	5′- TCCACCACCCTGTTGCTGTA -3′
FLG	5′- CAAATCCTGAAGAATCCAGATGAC -3′	5′- TGCTTGAGCCAACTTGAATACC -3′
LOR	5′- GTCTGCGGAGGTGGTTCCTCT -3′	5′- TGCTGGGTCTGGTGGCAGATC -3′
SPINK5	5′- CTGCTCCATGTGTGAGGTCTTC -3′	5′- CGTCCGTTTTTCCTGGATTTGCG -3′
SERPINB4	5′- CTGGGTGGAAAGTCAAACGAA -3′	5′- TGTCGTATCATTGCCAATAGTCC -3′
